# Multi-omics analysis revealed potential use of immunotherapy and CDK4/6 inhibitors in intimal sarcoma

**DOI:** 10.3389/fimmu.2025.1668537

**Published:** 2025-10-30

**Authors:** Bei Wang, Rongrong Chen, Huan Yin, Ziyi Chang, Yao Liu, Dingrong Zhong, Feng Li

**Affiliations:** ^1^ Medical Research Center & Department of Pathology, Beijing Chao-Yang Hospital, Capital Medical University, Beijing, China; ^2^ Department of Pathology, China-Japan Friendship Hospital, Beijing, China; ^3^ Geneplus, Beijing, China; ^4^ Medical Research Center & Department of Pathology, Beijing Chao-Yang Hospital, Beijing, China

**Keywords:** intimal sarcoma, angiosarcoma, cell cycle dysregulation, immunotherapy, tumor micro environment (TME)

## Abstract

**Background:**

Intimal sarcoma (IS) and angiosarcoma (AS), two rare yet highly aggressive vascular mesenchymal malignancies, present significant therapeutic challenges due to their scarcity, which underscoring the urgent need to investigate genetic alterations and tumor microenvironment (TME) features for novel therapeutic development.

**Methods:**

We performed integrated analysis of whole-exome sequencing (WES)/1021-gene panel sequencing, RNA sequencing, and immunohistochemistry (IHC) data from 31 IS and 35 AS patients to identify potential precision therapy.

**Results:**

Genomic profiling revealed 522 and 518 single nucleotide variants (SNVs) in the IS and AS cohorts, respectively. *TP53* mutations predominated in AS versus IS (15/35 vs 2/31, p < 0.001). Conversely, IS exhibited significantly more copy number variants (CNVs), particularly involving the *KDR/KIT/PDGFRA* locus (chromosome 4) and the *CDK4/MDM2* locus (chromosome 12) (*p < 0.001*). Strikingly, 25/31 (81%) IS patients harbored *CDK4* copy number gains or *CDKN2A/B* losses, compared to only 2/35 (6%) AS patients (*p < 0.001*). TME analysis revealed no significant inter-group differences overall; however, pulmonary artery IS specimens demonstrated substantial immune infiltration. Notably, reduced CD3^+^ T-cell density correlated with shorter survival (*p =0.029*). PD-L1 expression analysis (≥1% cutoff) showed positivity in 6/8 evaluable patients, including 3 with >50% tumor cell staining. Two IS patients receiving postoperative Sintilimab (PD-1 inhibitor) experienced prolonged survival (overall survival: 14+ and 56+ months, respectively).

**Conclusions:**

This study characterizes the distinct mutation landscape yet similar immune microenvironment of rare IS and AS. Given the frequent cell cycle dysregulation and the observed PD-L1 expression in a subset of patients, CDK4/6 inhibitors and PD-1/PD-L1 inhibitors warrant further clinical investigation for these patients.

## Introduction

Angiosarcoma (AS) is a rare histological subtype of soft tissue sarcoma that arises from endothelial cells of the blood or lymphatic vasculature. It frequently arises in the skin of the head and neck region, the breast, and may develop in almost any anatomic location (1–3). Intimal sarcoma is an even rarer and highly malignant tumor that primarily arises in large arteries and blood vessels (4). It extends along the intimal surface with multifocal intramural growth, most commonly involving the pulmonary artery, major systemic arteries (especially the aorta), and the heart. Characterized by a polypoid growth pattern within the lumen, pulmonary artery intimal sarcoma often invades the vascular wall and metastasizes distantly (5, 6).

The scarcity of AS and IS likely accounts for the severely restricted spectrum of available therapeutic interventions (7). Currently, AS and IS are generally treated with surgery, radiation, and chemotherapy. Surgical resection is the most effective treatment for both tumor types. Although patients undergoing curative resection show longer overall survival than those with incomplete resection (8–13), local and distant recurrences remain common. Notably, clinical outcomes have not markedly improved in decades despite aggressive therapeutic approaches (14).

In patients with advanced disease, chemotherapy can be effective but generally fails to provide durable clinical benefit (15, 16). It was reported doxorubicin-based chemotherapy failed in most IS patients (17), other cytotoxic agents such as tubulin inhibitor were still under investigation (18). Immune checkpoint blockade (ICB) represents a promising therapeutic approach across various soft tissue sarcomas. Impressive responses to ICB have been reported in a subset of AS patients (2, 19–28), with potential predictive markers including CD8+ lymphocytes expressing programmed cell death protein 1 (PD-1) and high tumor mutation burden (TMB) (29, 30). However, the efficacy of ICB in IS remains largely unknown. Recurrent alterations in the 12q12–15 region (encompassing *MDM2* and *CDK4*) and the 4q12 region (containing *PDGFRA*) in IS suggest that MDM2 and PDGFRA inhibition may constitute a viable treatment strategy (31–35). MDM2 inhibitors such as BI907828 Alrizomadlin (APG-115) and Milademetan (DS-3032) have demonstrated promising pharmacological effects in advanced preclinical models and early-phase clinical trials (12, 31, 36–41). Nevertheless, resistance to targeted therapies has been documented in IS (39). Furthermore, *MDM2/MDM4* amplifications have been associated with rapid disease progression, termed hyperprogressive disease (HPD), following ICB treatment in other contexts (42, 43). Consequently, whether ICB confers clinical benefit to IS patients requires further investigation.

In this study, we analyzed the clinical, genomic, and immune microenvironment characteristics of 31 patients with IS and 35 patients with AS to explore the potential for targeted therapy or immune checkpoint blockade immunotherapy in these rare but highly aggressive malignancies. We also present two cases of IS that benefited from ICB therapy, which may provide an additional treatment option for this devastating disease.

## Materials and methods

### Patient recruitment

This retrospective study analyzed 13 intimal sarcoma (IS) cases and 35 angiosarcoma (AS) cases identified from pathology archives between January 2017 and April 2025. Formalin-fixed paraffin-embedded (FFPE) samples from surgical resections or biopsies were collected for next-generation sequencing (NGS), immunohistochemistry (IHC), and fluorescence *in situ* hybridization (FISH) when available. Clinical data were retrieved from hospital records, and patients were followed for survival outcomes. The study was conducted in accordance with the Declaration of Helsinki and approved by the Institutional Ethics Review Board of the China-Japan Friendship Hospital (2023-KY-045). Individual consent for this retrospective analysis was waived.

Given the rarity of IS, we supplemented our cohort with 18 IS patients from a publicly available dataset (Memorial Sloan Kettering Cancer Center, MSKCC) as an independent cohort. The genomic data (cancer panel) and clinical information for these 18 cases were obtained via cBioPortal (44).

### IHC

Surgical and biopsy specimens underwent formalin fixation and paraffin embedding, followed by sectioning at 4.0 µm thickness. Sections were stained with hematoxylin and eosin (H&E) and immune-stained using the EnVision method, incorporating both negative and positive controls.

Primary antibodies were sourced as follows: CKpan (AE1/AE3), Vimentin (VMAB159), CD34 (EP88), Ki-67 (MIB-1), CD8 (SP16), and CD4 (EP204) from Beijing Zhong Shan - Golden Bridge Biological Technology Co., Ltd. (Beijing, China); SMA (1H4), CD3 (MX036), CD68 (Kp-1), MDM2 (IF2), and CDK4 (EP180) from Fuzhou Maixin Biotechnology Development Co., Ltd. (Fuzhou, China); PD-L1 (22C3) from Dako North America, Inc.; CD3 (L26) along with all secondary antibodies and reagents from Roche Diagnostics (Shanghai) Co., Ltd. Protein localization analyses were performed using established subcellular compartment criteria: nuclear staining for MDM2 and Ki-67; cytoplasmic staining for SMA, CKpan, CD3, CD8, CD4, CD68, and Vimentin; exclusive plasma membrane staining for PD-L1; combined cytoplasmic and plasma membrane staining for CD34; and cytoplasmic/nuclear dual localization for CDK4.

Immunostaining was scored as (+) when 1-10% of tumor cells were positive, (++) for 10% to 40% positive cells, and (+++) when positivity exceeded 40%. PD-L1 expression was assessed using the Tumor Proportion Score (TPS), defined as the percentage of viable tumor cells exhibiting partial or complete membrane staining at any intensity, with a positivity cutoff set at 1%.

### Fluorescence *in situ* hybridization

The *MDM2* (12q15) gene probe was employed to assess corresponding gene amplification in tumor cells using fluorescence *in situ* hybridization (FISH). The FISH probe kit was acquired from Guangzhou Amping Pharmaceutical Technology Co. Ltd. Procedures were performed in strict accordance with the manufacturer’s protocol. For interpretation, the average signals for *MDM2* and *CEP12* were enumerated across 50 tumor cells. *MDM2* amplification was defined as an *MDM2/CEP12* signal ratio ≥ 2.0.

### DNA extraction, targeted capture, and NGS

Genetic analysis was performed as previously described (45). In brief, serial sections of formalin-fixed paraffin-embedded (FFPE) tumor tissues were subjected to genomic DNA extraction using the QIAamp DNA Mini Kit (Qiagen, Valencia, CA). Histologically confirmed adjacent noncancerous tissue served as the control. Sequencing libraries were prepared using Illumina TruSeq DNA Library Preparation Kits (Illumina, San Diego, CA). Libraries were hybridized with custom-designed biotinylated oligonucleotide probes targeting either 1021 genes or the whole-exome sequencing (WES) panel (Integrated DNA Technologies, Inc.; **Supplementary Table 1**). Final libraries were sequenced on the GenePlus-Seq-2000 platform (GenePlus-Suzhou Institute).

### Sequencing data analysis and variant interpretation

The sequencing data were analyzed using default parameters (45). Adapter sequences and low-quality reads were removed. Clean reads were aligned to the human reference genome (hg19) using the Burrows-Wheeler Aligner (BWA; version 0.7.15-r1140). Single nucleotide variants (SNVs) were called using MuTect (version 1.1.4) and NChot. Small insertions and deletions (Indels) were identified with GATK. Somatic copy-number alterations (SCNAs) were detected using CONTRA (version 2.0.8). Significant SCNAs were defined as the log2 ratio of adjusted depth between tumor DNA and matched germline control DNA. All final candidate variants underwent manual verification in the Integrative Genomics Viewer (IGV). Targeted capture sequencing required a minimum mean effective depth of coverage of 300× for whole-exome sequencing (WES) and 500× for the 1021-gene panel. Variants were filtered to exclude synonymous alterations, known germline variants listed in dbSNP, and variants with a population frequency exceeding 1% in the Exome Sequencing Project.

### RNA sequencing and tumor-infiltrating lymphocyte subpopulation analysis

We performed RNA-Seq as previously described (46). Briefly, total RNA was extracted from tumor FFPE specimens using the RNeasy Mini Kit (Qiagen, Hilden, Germany) according to the manufacturer’s instructions. RNA integrity was assessed using the RNA Integrity Number (RIN) generated by the 2100 Bioanalyzer (Agilent Technologies, Santa Clara, CA, USA). RNA libraries were constructed with the NEBNext^®^ Ultra™ RNA Library Prep Kit (New England Biolabs, Beverly, MA, USA). Libraries were sequenced on the Geneplus-2000 platform (Geneplus, Beijing, China).

The relative fractions of 22 infiltrating immune cell types within each tumor tissue were determined using CIBERSORT (http://cibersort.stanford.edu/). The algorithm was executed using the LM22 signature matrix with 1,000 permutations. The evaluated tumor-infiltrating immune cell populations included naïve B cells, memory B cells, plasma cells, naïve CD4^+^ T cells, resting memory CD4^+^ T cells, activated memory CD4^+^ T cells, follicular helper T cells (Tfh), regulatory T cells (Tregs), CD8^+^ T cells, gamma delta T cells (γδ T cells), M0 macrophages, M1 macrophages, M2 macrophages, resting natural killer (NK) cells, activated NK cells, resting dendritic cells (DC), activated DC, monocytes, resting mast cells, activated mast cells, neutrophils, and eosinophils. For each tumor sample, the sum of the fractions for all evaluated immune cell types equaled 1 (47, 48).

## Results

### Characteristics of the patient cohort

The cohort comprised 66 cases (34 females, 32 males) with a mean age of 50.5 years (range, 17–83 years). The IS subgroup exhibited a higher proportion of female patients (20/31) compared to the AS subgroup (15/35). Tumor primary sites differed significantly, with IS predominantly involving the pulmonary artery and heart, and AS primarily involving the skin (**Table 1**).

**Table 1:** Characteristics of all the AS and IS patients.

Characteristics		All (n=66)	IS (n=31)	AS (n=35)
Gender	Female	34	20	14
	Male	32	11	21
Age at diagnosis		50.5 (17-83)	48.5 (19-83)	54.5 (17-81)
Primary site	Pulmonary artery	15	15	0
	Heart	11	8	3
	Skin	7	0	7
	Liver	4	0	4
	Spleen	4	0	4
	Breast	3	0	3
	Others	22	8	14

**Figure 1:**
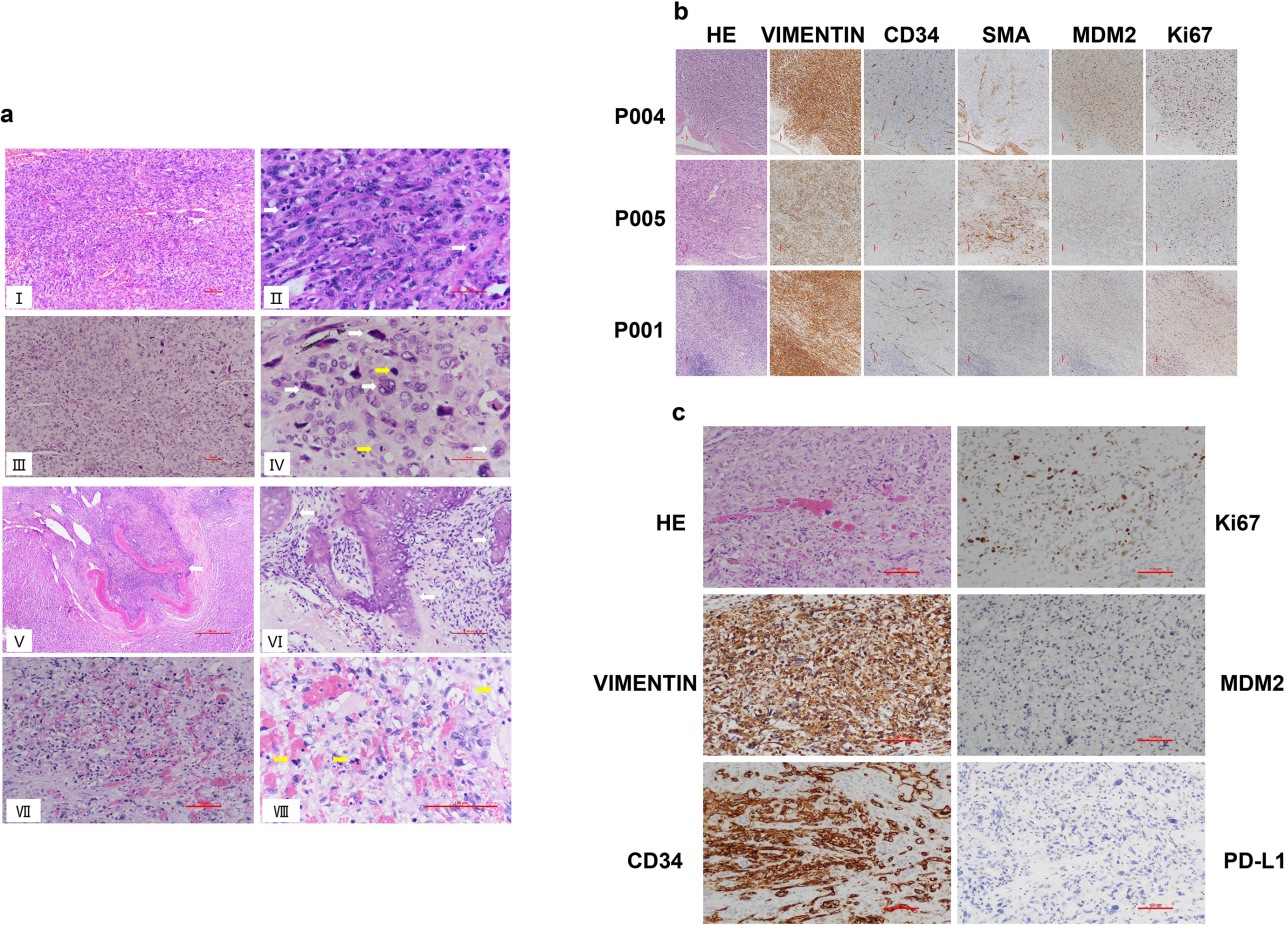
Histological analysis of intimal sarcoma and angiosarcoma. **(A)** Representative hematoxylin and eosin (H&E) staining of IS and AS cases. (I) Microscopically, the tumor tissue manifests as a poorly or undifferentiated malignant neoplasm of mesenchymal origin, predominantly composed of spindle cells within the tumor cell population. Scale bar: 100μm. (II) Spindle-shaped tumor cells are densely packed in fascicular formations akin to leiomyosarcoma, with mitotic figures readily discernible (white arrow). Scale bar: 50μm. (III) The tumor cells in some areas exhibit an epithelioid morphology, with relatively sparse cell density and prominent cellular pleomorphism. Scale bar: 100μm. (IV) High-power view showing highly atypical epithelioid and bizarre giant tumor cells (white arrows), with mitotic figures noted (yellow arrows). Scale bar: 50μm. (V) The tumor invades and destroys the vascular wall, with a small amount of residual vascular smooth muscle tissue visible (white arrow). Scale bar: 100μm. (VI) Region with osteosarcoma differentiation characterized by osteoid production (white arrow). Scale bar: 500μm. (VII) AS reveals variable tumor cell atypia, featuring anastomosing vascular channels lined by tumor cells and filled with numerous red blood cells. Scale bar: 100μm. (VIII) Tumor cells at high magnification exhibit abundant eosinophilic cytoplasm, vesicular nuclei with distinct nucleoli, and noted mitotic figures (yellow arrows). Scale bar: 100μm. **(B)** Immunohistochemical staining of Vimentin, MDM2, SMA, CD34 and Ki67 in representative cases of pulmonary artery intimal sarcoma (P004, P005, P001, 100×). Scale bar: 100μm. **(C)** Immunohistochemical staining of Vimentin, MDM2, CD34, Ki67 and PD-L1 in representative cases of hepatic angiosarcoma (200×). Scale bar: 100μm.

As pathological information for the 18 IS patients from the MSKCC cohort was unavailable (44), we focused on the 11 IS patients with detailed pathological analysis. Histologically, all tumors exhibited moderately to severely atypical spindle tumor cells. Epithelioid or giant tumor cells were present in 7 cases. Among the 8 cases with necrosis, these areas consistently featured severely atypical tumor cells, including epithelioid and bizarre giant tumor cells. Tumor cell density varied regionally. Some areas showed relatively sparse tumor cells with stromal myxoid degeneration, while others displayed densely packed spindle cells forming fascicles reminiscent of leiomyosarcoma. One case exhibited tumor invasion accompanied by destruction of the vascular wall. Osteosarcoma differentiation was identified in some cases (**Figure 1A**).

The tumor cells of IS and AS were diffusely positive for Vimentin (11/11, 100%) but negative for CKpan. Some IS tumor cells expressed MDM2 (8/11, 72.7%), SMA (3/11, 27.3%), and CD34 (2/11,18.2%). The Ki-67 proliferation index ranged from 30% to 90%, indicating a relatively high proliferation rate (**Figures 1B, C**).

Archived FFPE samples were utilized for tumor genomic DNA and RNA extraction when available. For the 48 patients from our center, all provided sufficient DNA for subsequent NGS analysis, with 5 undergoing whole-exome sequencing (WES) and the remaining 43 analyzed using a 1021-gene panel. The 18 IS patients from the MSKCC cohort underwent sequencing with IMPACT341/410/468 panels. Additionally, qualified RNA was obtained from 17 patients (11 IS and 6 AS) for RNA sequencing.

### Genomic profiling showed significant difference between IS and AS

**Figure 2:**
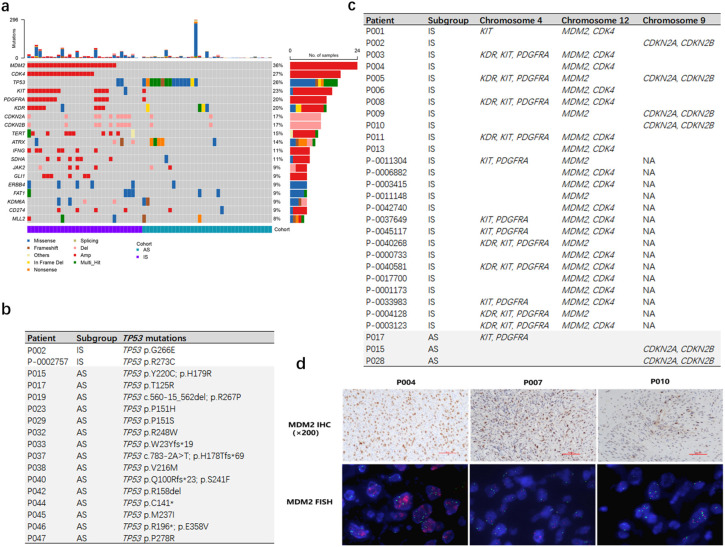
Genomic mutation analysis of IS and AS patients. **(A)** The genetic landscape and clinical characteristics of 66 patients. The mutation landscape shows the variation of the top 20 genes with the highest mutation frequency in tumors. Each square shows the mutation of each sample provided by the patient in one gene. Different colors denote 8 types of mutations. **(B)** IS patients had a lower proportion of *TP53* mutations. **(C)** IS patients had a higher proportion of copy number amplification in *MDM2*, *CDK4*, *KIT*, *PDGFRA*, *KDR* genes. **(D)** FISH analysis of *MDM2* CNV in patients (P004, P007, P010) confirmed true positive of *MDM2* in P004, but negative in P007 and P010. (MDM2 IHC, 200×).

Somatic mutations were identified in all 66 patients, with 522 single nucleotide variants (SNVs) detected in the 31 IS patients and 518 SNVs in the 35 AS patients. Copy number variants (CNVs) totaled 268 in IS patients and 65 in AS patients. Collectively, the most frequently mutated genes were *MDM2* (36%, 24/66)*, CDK4* (27%, 18/66)*, TP53* (26%, 17/66)*, KIT* (23%, 15/66) and *PDGFRA* (20%, 13/66) (**Figure 2A**).

*TP53* mutations were significantly more prevalent in AS patients compared to IS patients (15/35 vs 2/31, p < 0.001, **Figure 2B**). Conversely, amplifications of *KDR*, *KIT* and *PDGFRA* on chromosome 4, as well as *CDK4* and *MDM2* on chromosome 12, were enriched in IS patients (p < 0.001; **Figure 2C**).

Moreover, *CDK4* copy number gains or *CDKN2A/CDKN2B* losses were observed in 25 of 31 IS patients but only in 2 of 35 AS patients (p < 0.001; **Figure 2C**). This cell cycle dysregulation may underlie the elevated Ki-67 index 30%- 90% in IS patients (**Figure 1B**).

Overexpression and amplification of *MDM2* constitute an important characteristic in IS. Further analysis was performed on IS patients lacking *MDM2* CNV within our cohort. Among the four patients with available MDM2 IHC staining results, two were MDM2 IHC ++ (P003, P007) and two were MDM2 IHC + (P002, P010). Subsequent *MDM2* FISH analysis on samples P007 and P010 also showed no *MDM2* copy number gain in either specimen. (**Figure 2D**, **Supplementary Table S1**). The observed discrepancies among IHC, NGS-CNV, and FISH results are likely attributable to the low tumor cell fraction in these samples.

### Tumor microenvironment analysis showed similar features in IS and AS

**Figure 3:**
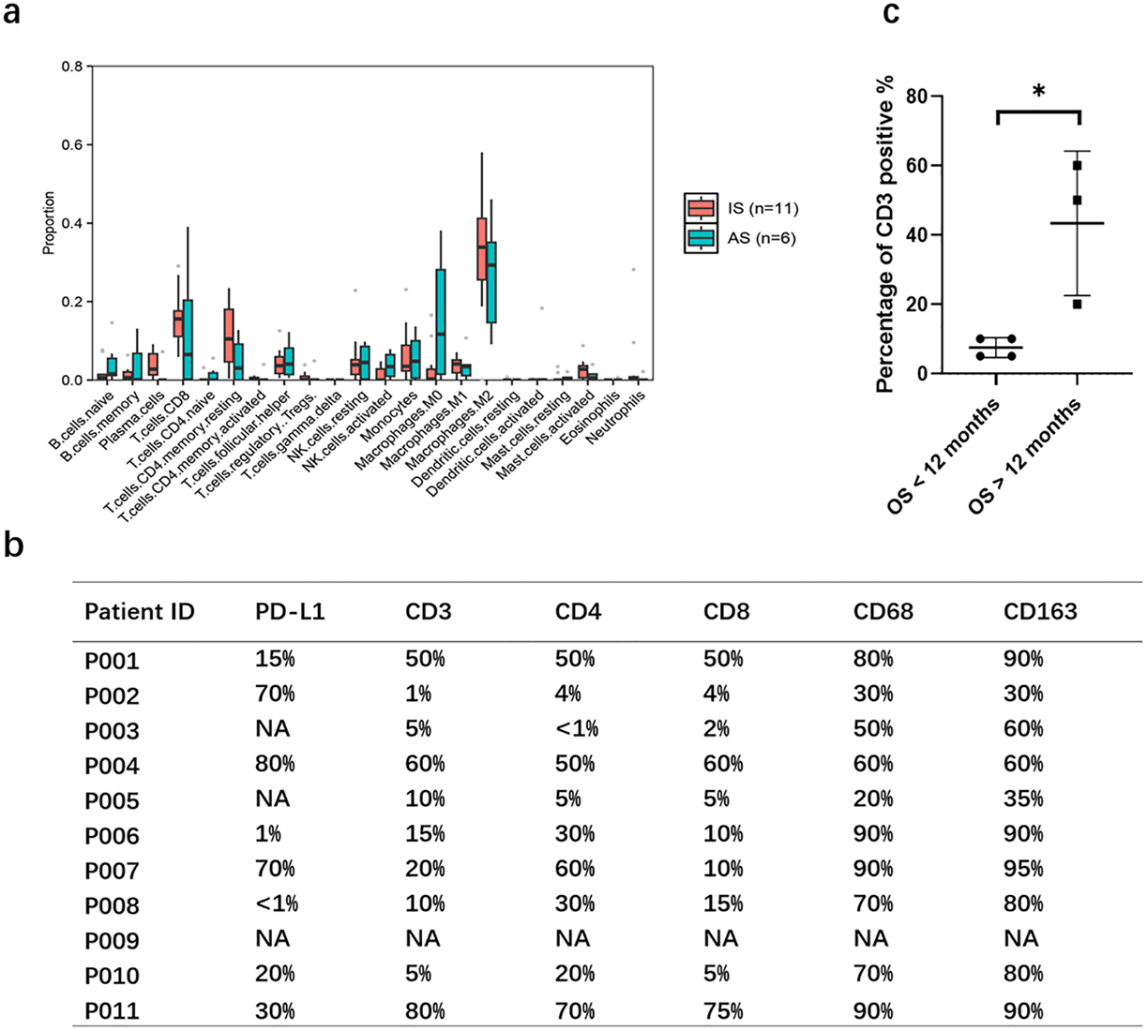
Characteristics of the immune microenvironment. **(A)** Analysis of 22 immune cell infiltration according to CIBERSORT algorithm showing the percentage of immune cells in each sample. **(B)** PD-L1 expression and immune cell infiltration of pulmonary artery intimal sarcoma. **(C)** Percentage of CD3 positive cells was lower in patients with overall survival (OS) less than 12 months. Statistical significance of differences between the groups was calculated with Mann Whiney U test. P-value< 0.05 is considered statistically significant. *p < 0.05.

Given the striking differences in genomic profiling between the IS and AS cohorts, we further investigated characteristics of the tumor microenvironment in these groups. Using the CIBERSORT algorithm to calculate the abundance of 22 immune cell types within each sample, we found no significant difference in immune infiltration between the IS and AS cohorts (**Figure 3A**).

We next examined PD-L1 expression via IHC in IS patients. Among eight patients with sufficient FFPE samples, PD-L1 expression was detected in six, with three patients (P002, P004, P007) exhibiting strong positivity (70%, 80%, and 70%, respectively; **Figure 3B**, **Supplementary Figure S1**). Although the small sample size (n=8) limits broad conclusions, this preliminary finding suggests that PD-L1 expression is a clinically relevant feature in a considerable proportion of IS patients and may indicate a potential for response to ICB. Given the observed abundant immune cell infiltration- including lymphocytes, plasma cells, and histiocytes, with some lymphocytes forming stromal clusters- we characterized the immune microenvironment using the following markers: CD3+ for total T cells, CD4+ for helper T cells, CD8+ for cytotoxic T cells, CD68+ for macrophages, and CD163+ for monocytes/macrophages. Analysis revealed median immune cell infiltration levels of 13% CD3+ (range, 1–80%), 10% CD8+ (range, 2–75%), 30% CD4+ (range, <1–70%), 70% CD68+ (range, 20–90%), and 80% CD163+ (range, 30–90%) (**Figure 3B**, **Supplementary Figure S2**). Notably, patients who died within 12 months post-surgery (n=4, P003, P005, P008, P010) exhibited significantly lower CD3+ infiltration compared to those surviving beyond 12 months (n=3; P001, P004, P007; median 7.5% vs. 70%, P=0.029; **Figure 3C**).

### Efficacy of immune checkpoint inhibitor in 3 IS patients

Among the 13 IS patients treated at our center, eight underwent pulmonary endarterectomy and one received wedge resection of the left lower lobe. Three patients died postoperatively on day 5, month 4, and month 11, respectively. Of the five surviving surgical patients, two maintained disease-free survival at 11–14 months. Two experienced relapse, while one patient relapsed at 12 months but remained alive for over 56 months with maintenance therapy comprising sintilimab (anti-PD-1 immunotherapy) and anlotinib (targeted therapy). Two patients did not undergo pulmonary artery tumor resection: one due to inoperability (adrenal metastasis) and another who refused surgery and succumbed to disease progression at 5 months (**Table 2**).

**Table 2:** Characteristics of IS patients.

Patient ID	Site	Surgery	Adjuvant therapy	Outcome
P001	PT, LPA, RPA	PEA	ICI	alive, progress free survival 14 months
P002	RPA, lung, adrenal gland	Bronchoscopy	CT	alive, followed for 5 months
P003	RPA, chest, vena azygos	Bronchoscopy	—	died 5 months after diagnosis
P004	PT, LPA, RPA, lung	PEA, right upper lobectomy	CT	alive, progress free survival 18 months
P005	LPA	Wedge resection of left lower lobe of lung	CT + AAT + ICI	died 11 months after surgery
P006	PT, LPA, RPA, PV, RVOT	PEA	CT + RT	relapsed 2 months after PEA
P007	PT, LPA, RPA	PEA	CT + AAT + ICI	relapsed 12 months after PEA, alive over 56 months
P008	PT, LPA, RPA, PV	PEA	CT	died 4 months after PEA
P009	PT, LPA	PEA	—	lost to follow-up
P010	PT, LPA, RPA	PEA	—	died 5 days after PEA
P011	PT, LPA, RPA	PEA	CT	relapsed 2 months after PEA

PT, pulmonary trunk; LPA, left pulmonary artery; RPA, right pulmonary artery; PV, pulmonary valve; RVOT, right ventricular outflow tract; PEA, Pulmonary endarterectomy; CT, Chemotherapy; ICI, Immune checkpoint inhibitor; AAT, antiangiogenic therapy; RT, radiotherapy.

**Figure 4:**
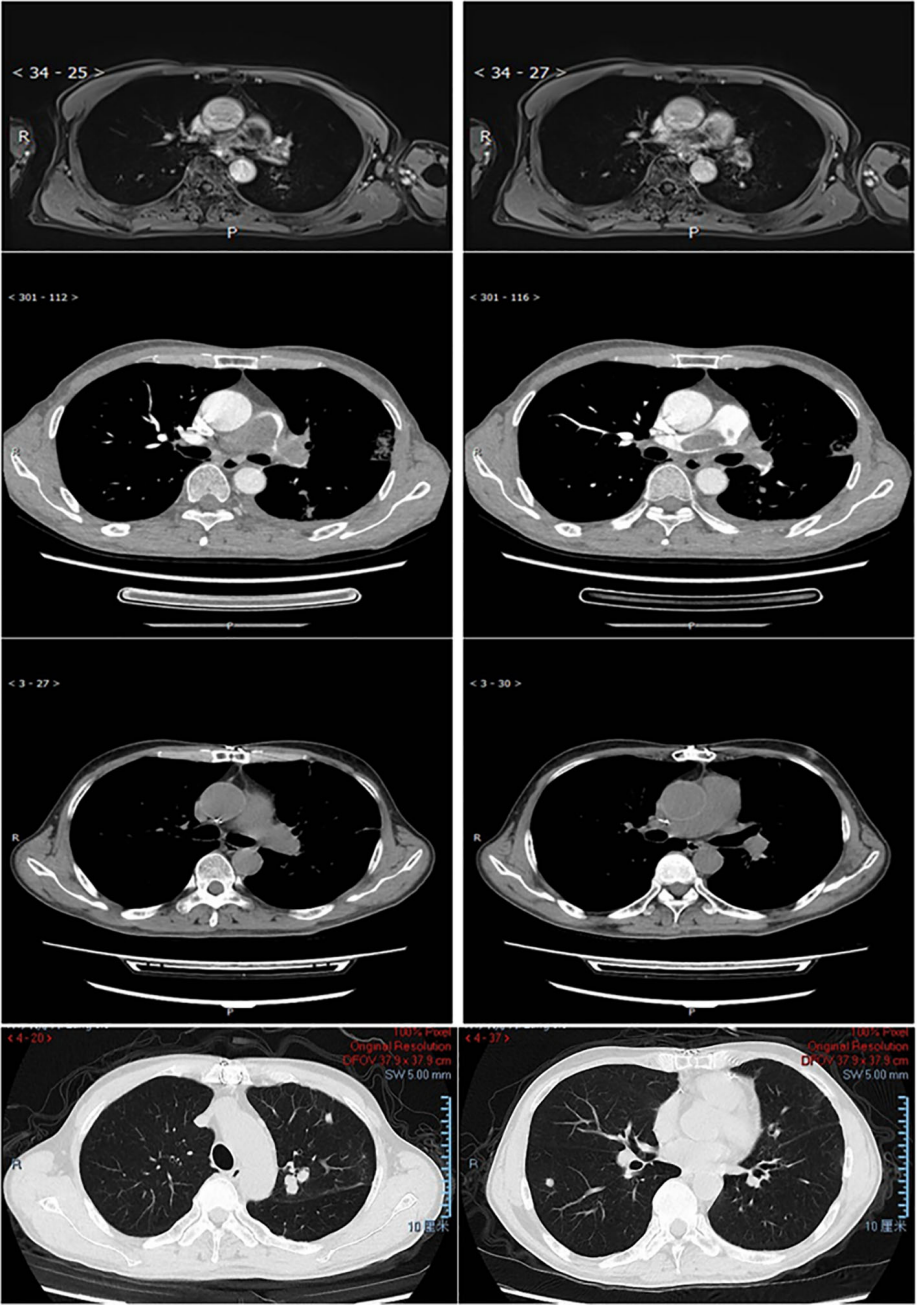
MRI/CT scan of the lung in patient P007. Row 1 (MRI) 2 (CTA), pulmonary artery intimal sarcoma involved PT, LPA, and RPA at diagnosis. Row 3-4, 12 months after PEA the patient relapsed with tumor involved left upper lobe, right lower lobe, and multiple little nodules in the peripheral lung.

Three patients received immune checkpoint inhibitor (ICI) therapy during their disease course. Patient P001, an adult patient with pulmonary artery intimal sarcoma involving the pulmonary trunk (PT), left pulmonary artery (LPA), and right pulmonary artery (RPA), received adjuvant sintilimab following pulmonary endarterectomy (PEA) and has remained progression-free for over 14 months to date. The tumor exhibited 15% PD-L1 expression and strong MDM2 positivity, with an *MDM2* copy number gain (CNG) of 9.15. Patient P007, an adult patient also diagnosed with pulmonary artery intimal sarcoma involving the PT, LPA, and RPA, experienced tumor recurrence 12 months post-PEA. This patient subsequently underwent 4 cycles of combined therapy including chemotherapy, the antiangiogenic agent anlotinib, and sintilimab, followed by sintilimab maintenance therapy, achieving sustained tumor control for over 56 months since initial diagnosis (**Figure 4**). Patient P005, an adult patient with pulmonary artery intimal sarcoma confined to the LPA, underwent wedge resection of the left lower lobe. Despite negative PD-L1 expression, adjuvant therapy comprising combined chemotherapy, antiangiogenic therapy, and an ICI was administered; however, the patient died 11 months post-surgery. The tumor was MDM2-positive, with an *MDM2* CNG of 5.37.

## Discussions

We performed comprehensive genetic profiling of intimal sarcoma (IS) and angiosarcoma (AS) by integrating WES/1021-panel, RNA-seq, and IHC data from our cohort with IS sequencing data from MSKCC cohorts. Our analysis demonstrated that IS and AS represent distinct sarcoma subtypes but with similar tumor microenvironment: copy number variations (CNVs) were highly enriched in IS, whereas *TP53* mutations were predominant in AS. Thus, targeted therapies should be different for IS and AS, while immunotherapy might be of similar efficacy.

In terms of targeted therapy, consistent with prior reports in IS, the most frequent genetic alterations were copy number variations (CNVs) at 12q12-15 (encompassing *MDM2* and *CDK4*) and 4q12 (encompassing *KDR*, *KIT* and *PDGFRA*) (6, 37, 38, 49–51). Thus, MDM2 and PDGFRA inhibition constitute a viable treatment strategy which warrant further clinical trials (12, 31–34, 36–41). In our study, we also observed an increased incidence of*CDKN2A/CDKN2B* copy number loss at 9p21 in IS patients lacking *CDK4* copy number gain. Collectively, these aberrations in cell cycle regulators may underlie the elevated Ki-67 indices (30%–90%) characteristic of IS tumors. Given that the phase II trial of the CDK4/6 inhibitor palbociclib in advanced sarcoma, which selected patients based on*CDK4* mRNA expression relative to *CDKN2A*, achieved its primary endpoint (52), and the successful combination of ribociclib (CDK4/6 inhibitor) with everolimus (mTOR inhibitor) in advanced dedifferentiated liposarcoma and leiomyosarcoma (53), other CDK4/6 inhibitors warrant further investigation in IS.

Regarding immune checkpoint blockade (ICB), while several studies have reported impressive responses in a subset of AS patients (2, 19–28), clinical activity of ICB in intimal sarcomas was only reported in separated cases and its efficacy remains poorly characterized (54, 55). C Park et al. classified IS patients into CNV-high (CNV-H) or MSI-H-like subtypes based on copy number variation (CNV) enrichment (featuring frequent *CDK4/MDM2* amplifications) or predominant *MLH1* mutations, respectively. They described two MSI-H-like patients treated with pembrolizumab, one achieving complete remission lasting 2 years and the other exhibiting disease control for 6.5 months (56). In our cohort, we identified mutations (*MLH1* p.P581L, *MSH6* p.F1088Lfs*5, *MLH3* p.T730Qfs*4), but all three occurred in a single patient (P006), who also had *MDM2* copy number gain. This co-occurrence precluded definitive classification into the CNV-H or MSI-H-like subtypes within our IS cohort. Notably, however, we observed generally similar tumor microenvironments between AS and IS patients, suggesting potential ICB efficacy in a subset of IS patients as well. Our cohort included 13 IS patients of varying ages, with a predominance of females. Surgical resection remained the primary treatment for operable lesions, with chemotherapy (with or without antiangiogenic therapy) or radiation therapy serving as common adjuvant approaches. Reflecting the overall poor prognosis of pulmonary artery intimal sarcoma, three patients received immunotherapy to prevent recurrence or treat recurrent disease. In these three patients, survival duration appeared prolonged, and hyperprogression was not observed despite *MDM2* copy number gain-a factor implicated in hyperprogression following ICB in other solid malignancies (42, 43). We speculate that the *MDM2* copy number gain in these patients represents chromosome 12 polysomy rather than specific *MDM2* amplification, as the *IFNG* gene (located at 12q15) exhibited similar copy number changes. Combined with the PD-L1 expression observed in a subset of our IS cohort and another study (57), these preliminary data suggest that the potential value of immunotherapy in this aggressive disease warrants further investigation in larger, prospective cohorts.

This study has several limitations: (1) Due to its retrospective nature, we were unable to collect complete therapeutic details, including imaging data, for all patients, and not all patients underwent PD-L1 testing or whole-exome sequencing (WES) due to insufficient tumor sample availability; (2) The rarity of this disease limited enrollment to a small cohort of immunosuppressed (IS) patients, necessitating the inclusion of MSKCC patients; (3) The immunohistochemical analysis in this study was primarily descriptive and semi-quantitative. Future studies with larger patient cohorts are needed to perform robust quantitative assessments of protein expression and immune cell infiltration to validate our findings.

In conclusion, we systematically analyzed the clinical characteristics, pathogenic mechanisms, molecular mutation landscape, and immune microenvironment of rare intimal sarcoma and angiosarcoma. Our data reveal frequent cell cycle dysregulation and identify PD-L1 expression in a subset of these tumors. These findings suggest that CDK4/6 inhibitors and PD-1/PD-L1 inhibitors, may represent promising therapeutic strategies worthy of further investigation.

## Data Availability

The data presented in this study are deposited in the National Genomics Data Central (NGDC) repository, accession number HRA013989.

## Supplementary material

Supplementary Figure 1 Immunohistochemical staining of PD-L1 (22C3) in representative patients (P005, P010, P011, P004), with PD-L1 ranging from 0% to 80% (200×).

Supplementary Figure 2 Immunohistochemical staining of immune cells in representative patients (P004, P001, P010). Column 1, HE, 400×;column 2, CD3, 200×;column 3, CD4, 200×;column 4, CD8, 200×; column 5, CD68, 200×. Row 1, patient P004 showed high lymphocyte densities, row 2 patient P001 showed high lymphocyte densities with focal lymphocyte infiltration, row 3 P010 showed low infiltration of immune cells.
