# Supplementary figures and images for: Multi-omics analysis revealed potential use of immunotherapy and CDK4/6 inhibitors in intimal sarcoma
Source: Front Immunol. 2025 Oct 30;16:1668537. doi: 10.3389/fimmu.2025.1668537 (PMC12611966; doi:10.3389/fimmu.2025.1668537)

Figure S1

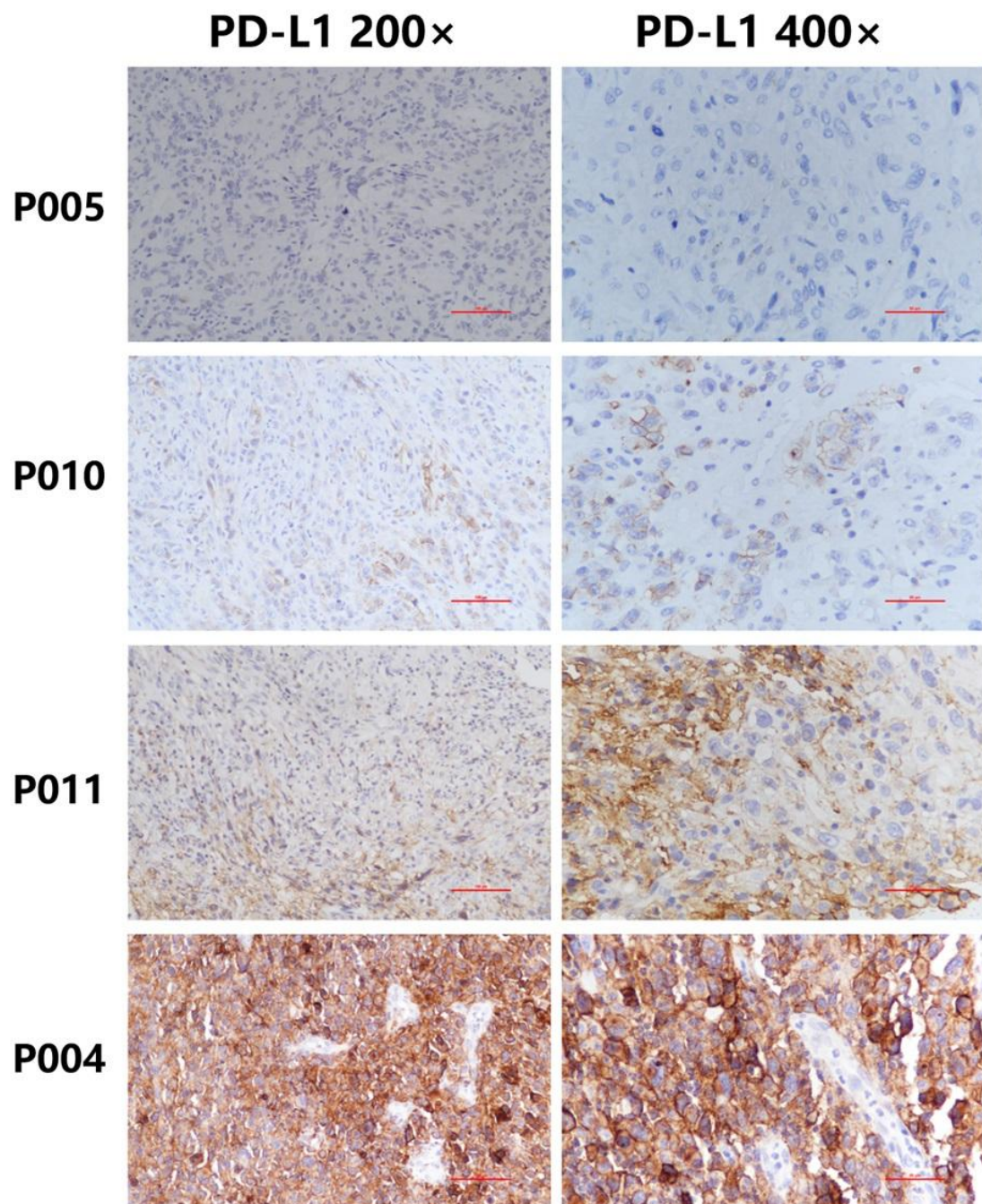

Figure S2

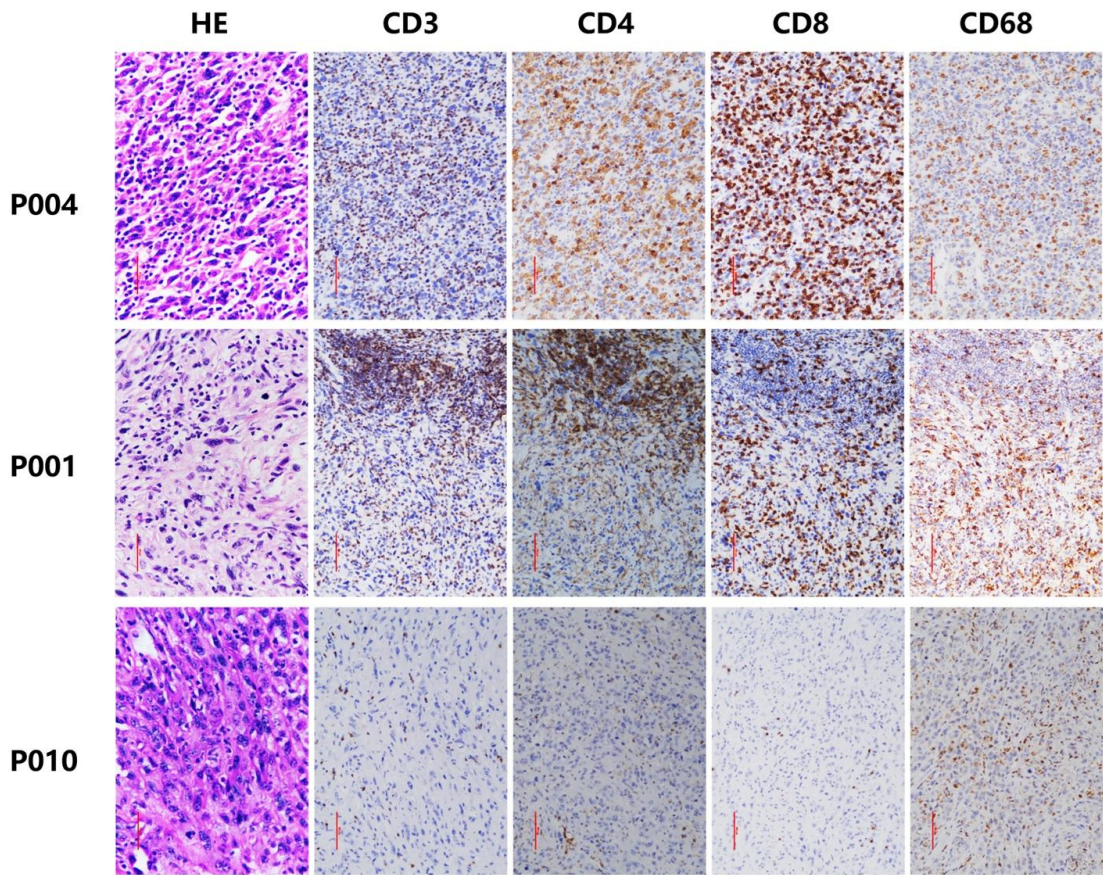

Supplement: Supplementary Figure 1 — Immunohistochemical staining of PD-L1 (22C3) in representative patients (P005, P010, P011, P004), with PD-L1 ranging from 0% to 80% (200×). [file DataSheet1.pdf]
