# Supplementary material for: Multi-omics analysis revealed potential use of immunotherapy and CDK4/6 inhibitors in intimal sarcoma
Source: Front Immunol. 2025 Oct 30;16:1668537. doi: 10.3389/fimmu.2025.1668537 (PMC12611966; doi:10.3389/fimmu.2025.1668537)
Supplement: Supplementary file 2 [file Table1.docx]

Table S1 IHC and FISH analysis of MDM2 and CDK4

| Patient ID | Ki-67 IHC | MDM2-CNG | MDM2-IHC | MDM2-FISH | CDK4-CNG | CDK4-IHC | CDK4-FISH |
| --- | --- | --- | --- | --- | --- | --- | --- |
| P001 | 30% | 9.15 | +++ | NA | 7.89 | ++ | NA |
| P002 | 80% | 2.00 | + | NA | 2.00 | + | NA |
| P003 | 50% | 2.00 | ++ | NA | 8.45 | +++ | NA |
| P004 | 40% | 25.80 | +++ | positive | 6.12 | + | positive |
| P005 | 40% | 5.37 | ++ | NA | 2.00 | ++ | NA |
| P006 | 60% | 3.35 | ++ | negative | 3.69 | +++ | negative |
| P007 | 50% | 2.00 | ++ | negative | 2.00 | ++ | positive |
| P008 | 40% | 11.43 | +++ | positive | 5.63 | +++ | positive |
| P009 | 40% | 7.05 | NA | NA | 2.00 | NA | NA |
| P010 | 40% | 2.00 | + | negative | 2.00 | ++ | negative |
| P011 | 50% | 4.80 | +++ | NA | 5.92 | + | NA |
